# Patient-experienced burden of treatment in patients with multimorbidity – A systematic review of qualitative data

**DOI:** 10.1371/journal.pone.0179916

**Published:** 2017-06-23

**Authors:** Michael Rosbach, John Sahl Andersen

**Affiliations:** The Research Unit for General Practice and Section of General Practice, Department of Public Health, University of Copenhagen, Copenhagen, Denmark; TNO, NETHERLANDS

## Abstract

**Objective:**

To synthesize existing qualitative literature on patient-experienced burden of treatment in multimorbid patients.

**Methods:**

A literature search identified available qualitative studies on the topic of burden of treatment in multimorbidity and meta-ethnography was applied as method. The authors’ original findings were preserved, but also synthesized to new interpretations to investigate the concept of the burden of treatment using the Cumulative Complexity Model.

**Results:**

Nine qualitative studies were identified. The majority of the 1367 participants from 34 different countries were multimorbid. The treatment burden components, experienced by patients, were identified for each study. The components financial burden, lack of knowledge, diet and exercise, medication burden and frequent healthcare reminding patients of their health problem were found to attract additional attention from the multimorbid patients. In studies conducted in the US and Australia the financial burden and the time and travel burden were found most straining to patients with deprived socioeconomic status.

The burden of treatment was found to be a complex concept consisting of many different components and factors interacting with each other. The size of the burden was associated to the workload of demands (number of conditions, number of medications and health status), the capacity (cognitive, physical and financial resources, educational level, cultural background, age, gender and employment conditions) and the context (structure of healthcare and social support).

Patients seem to use strategies such as prioritizing between treatments to diminish the workload and mobilizing and coordinating resources to improve their ability to manage the burden of treatment. They try to routinize and integrate the treatment into their daily lives, which might be a way to maintain the balance between workload and capacity.

**Conclusions:**

Healthcare providers need to increase the focus on minimizing multimorbid patients’ burden of treatment. Findings in this review suggest that the weight of the burden needs to be established in the individual patient and components of the burden must be identified.

## Introduction

Half of the adult population have chronic conditions[1], and as the treatment for chronic conditions has improved, the life expectancy has increased and continues to do so[2]. As a result multimorbidity, the co-existence of two or more chronic conditions, has emerged as a new major health concern.

A review investigating the definition of multimorbidity in the scientific literature showed that while diseases were included in all definitions, risk factors were often (87%) and symptoms less often (62%) included[3]. Exactly which diseases, risk factors and symptoms to include in the definition is up for debate, and that partly explains why a review including studies with different definitions of multimorbidity from countries all over the world shows prevalences between 12,9% and 95,1%. However, the review does show that the main part of prevalences are above 20%, increasing with age[4].

A cross-sectional study conducted in Scotland in 2012 found multimorbidity to be associated with socioeconomic status as the onset of multimorbidity occurred 10–15 years earlier in people living in the most deprived areas compared with the most affluent[5].

The increasing prevalence of multimorbidity has several adverse consequences. Multimorbidity is known to result in a decreased quality of life[6], a higher mortality rate[7] and increased healthcare utilization and cost in primary and secondary care[8]. Patients who have chronic conditions and multimorbidity experience a variety of symptoms, but in addition to this burden of illness, they are also affected by the burden of treatment (BoT). The BoT has only recently started attracting attention and includes the challenges of everything patients do to manage their conditions. It has been suggested that patients suffering from multimorbidity and an excessive BoT might not adhere to prescribed medical treatment [9, 10]. This may in some cases pose a problem since poor adherence to certain evidence-based pharmacotherapy is known to lead to greater risk of hospitalization and mortality[11, 12].

### The Cumulative Complexity Model

Several different models and theories have been used to describe the BoT[13–15]. Shippee et al. created in 2012 the Cumulative Complexity Model[13] by conducting a narrative literature review. The model states that clinical and social factors accumulate and add to a certain workload on the patient, balanced by the capacity (cognitive, physical and financial resources) of the patient. This balance results in healthcare access, utilization and self-care, which loops back and affects the workload and capacity. All of the above mentioned factors might have an impact on the health outcome of the patient. If a worsened health outcome encourages the healthcare provider to adjust the treatment and increase the size of the BoT, this might loop back and affect workload and capacity with risk of creating a vicious circle. The model will be used for understanding findings in this review.

As accounted for, the BoT poses an increasing problem in multimorbid patients and a deeper understanding of the BoT is needed. Several qualitative studies addressing the BoT from the perspective of patients with multimorbidity have been conducted. A review synthesizing these studies will provide a broader image of the BoT and until now, no such review has been conducted. Therefore, the purpose of this paper is to investigate the BoT in multimorbid patients by systematically reviewing empirical qualitative research to answer following questions:

### Research questions

1. Which components form the burden of treatment in the view of patients with multimorbidity?
2. Which components attract additional attention from the multimorbid patients?
3. How is the patient-experienced burden of treatment in patients with multimorbidity conceptualized in the included studies?

## Methods and material

Meta-ethnography developed by Noblit and Hare[16] is a method of seven steps to synthesize qualitative data. It has been suggested that meta-ethnography is particularly suitable when looking at individuals’ experiences[17], and the method has been successfully used in other reviews to understand medicine-taking and patients’ experience of diabetes and diabetes-care[18–20]. The strength of this approach lies in its ability to synthesize qualitative studies and produce new interpretations, while still preserving the interpretations of the original studies. Meta-ethnography is chosen as the best method for conducting this systematic review as it investigates qualitative data on patient-experienced BoT.

Step 1, *getting started*: Relevant literature search regarding multimorbidity and BoT was conducted with the assistance of a research librarian at the university library, and the research questions were formulated.

Step 2, *confirming initial interest*: The search for relevant literature included PubMed, Embase and PsycINFO and was done in accordance to the PRISMA guidelines[21]. The goal of the search was to find literature on the topic”burden of treatment” combined with the topic”multimorbidity” or the topic”general practice”. “General practice” was added to identify studies of BoT in a population recruited from general practice, likely to contain a high proportion of patients with multimorbidity[22]. The search in PubMed was built of three components:

1. "Burden of treatment" OR "treatment burden" OR "burden of care"
2. Multimorbid* OR”comorbidity"[Mesh] OR comorbid* OR”chronic disease"[Mesh] OR”chronic disease*” OR “chronic conditions" OR "chronic illness*"
3. ”General practice"[Mesh] OR "general practice" OR "family practice" OR "primary care" OR "primary health care"[Mesh] OR "primary health care" OR "primary healthcare"

The investigation included a search for the combinations of (1 AND 2) or (1 AND 3) in June 2016. Similar search was conducted in Embase and PsycINFO.

Abstracts and titles were screened and records not concerning patients’ BoT or only smaller parts of it, like the financial burden or polypharmacy, were excluded. Reviews and records describing theories and models without empirical data were also excluded. This left 39 articles, which were assessed in full text for eligibility.

**Fig 1:**
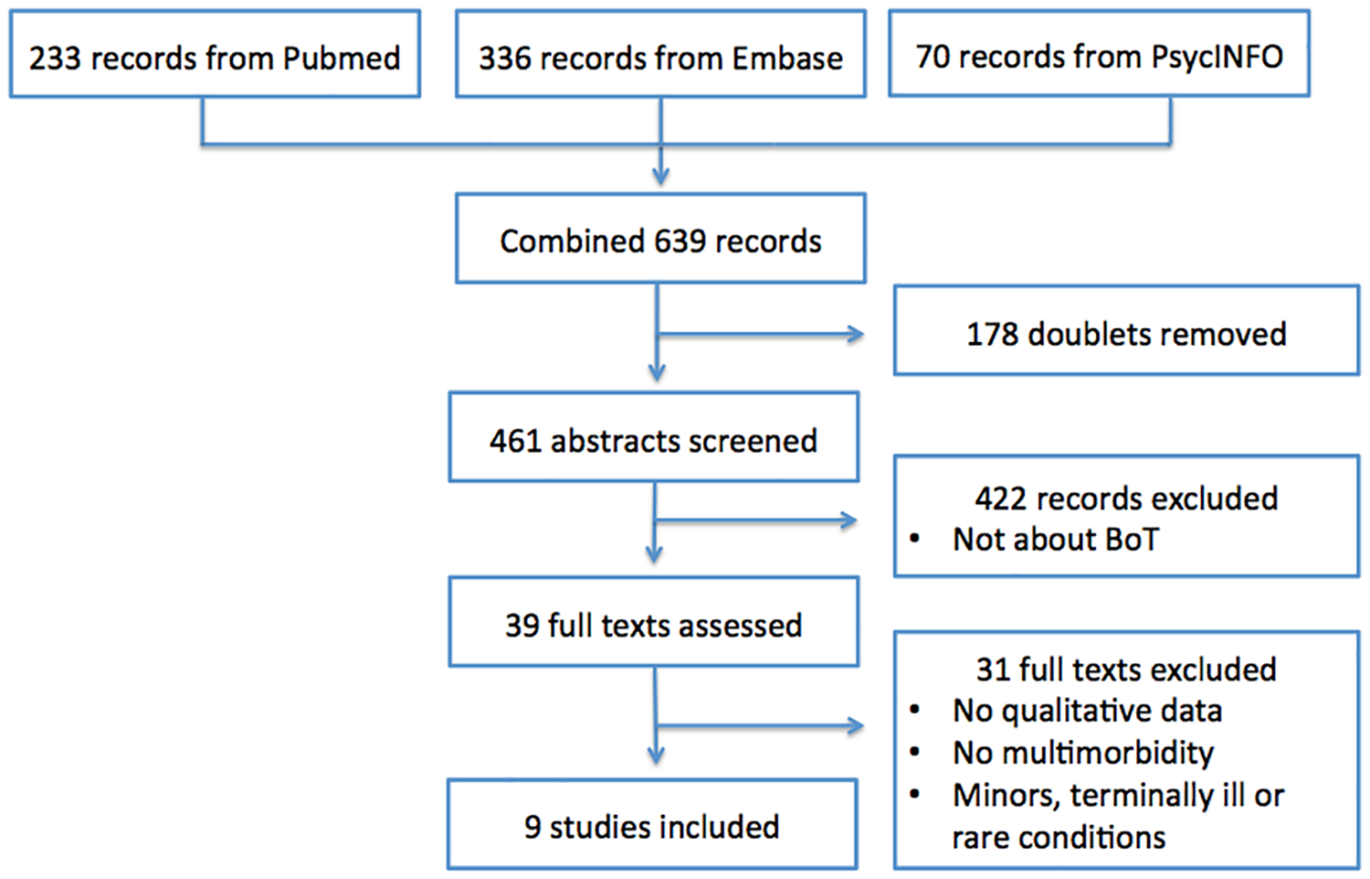
Flow diagram of the search for literature.

Only studies investigating self-reported BoT in a population, in which the main proportion of the participants had multimorbidity, were included. Articles not concerning multimorbid patients or only concerning minors, terminally ill patients or rare conditions were excluded. Reference lists were searched to check if any substantial articles were missing, but none additional were found. Fig 1 shows the flow diagram of the search.

The quality of the included studies were assessed according to a checklist developed by Malterud[23] covering aim of the study, reflexivity, methods and design, data collection and sampling, theoretical framework, methods of analysis, results, discussion as well as presentation.

Step 3, *reading studies and extracting data*: Data, objectives, methods, theoretical perspectives and working definitions of the BoT of the studies were extracted and illustrated in Tables 1 and 2.

**Table 1:** Details of included studies.

Authors	Year	Country	Participants					QA
			Who	N	Age (mean)	MM	Recruited from	
Tran, Barnes et al.[24]	2015	34 diffe-rent	Adult participants with at least one chronic condition	1053	35–57 (47)	63%	34 different mainly Western countries through the internet	27
Sav et al.[25]	2013	Australia	People with chronic conditions and their unpaid carers	85	16–83 (57)	88%	Four culturally and geographically diverse districts	28
Noël et al.[26]	2004	US	Patients having two or more chronic illnesses	60	30–89	100%	Eight primary care clinics within the Veterans Health Administration—Four in large urban metropolitan settings, four in rural communities	24,5
Eton et al.[27]	2015	US	1^st^ round: Patients with one or more chronic condition and complex self-care 2^nd^ round: Diabetic, heart failure and kidney failure patients	50	25–85 (56)	98%	Mayo Clinic Rochester (specialized treatment) and Hennepin County Medical Center Minneapolis (large safety-net hospital)	28,5
Ridgeway et al. [28]								28,5
Gallacher et al.[29]	2011	UK	Patients with chronic heart failure and comorbidities	47	45–88 (73)	100%	Primary care	25
Kahn et al.[30]	2014	US	Low income US primary care patients with chronic kidney disease	34	(62)	>94%	Two primary care (safety net) practices in Buffalo, a low-income African-American area which constitutes a”Health Professional Shortage Area”	26,5
Tran, Montory et al.[31]	2012	France	Patients with at least one chronic condition	22	53–76 (70)	NA	Department of internal medicine of a French hospital and a general practitioner clinic in Paris	27,5
Bayliss et al.[32]	2003	US	Adults with two or more self-reported conditions	16	31–70+ (61)	100%	Urban family practices in the Carenet network (serving disadvantaged populations) in Denver, Colorado	26

MM = Proportion of participants with multimorbidity in %. QA = Quality assessment score. Maximum score 29 [23].

**Table 2:** Details of included studies.

Authors	Objective	Methods	Theoretical perspective and working definition of BoT
Tran, Barnes et al.	To describe and classify the components of the burden of treatment for patients with chronic conditions	Open-ended questions in an online survey in English, French and Spanish. Content analysis (grounded theory approach). Reinert’s automatic textual analysis. Taxonomy, Bradley et al.’s method	Questionnaire developed after literature review, reviewed by seven experts. BoT defined as “work of being a patient” on functioning and well-being
Sav et al.	To explore treatment burden among people with a variety of chronic conditions and comorbidities	Semi-structured interviews face to face or over phone. Analyzed using iterative thematic approach and constant comparison method (grounded theory analysis)	The study was guided by the interpretive social paradigm, described by Neuman[33]. BoT defined as consequences patients and their families experience as a result of undertaking or engaging in treatment
Noël	To explore the collaborative care needs and preferences in primary care patients with multiple chronic illnesses	Semi-structured interviews in focus groups. Descriptive codes were grouped to generate broader themes. Patterns, interrelationships and overarching categories were discerned among the themes	Results grouped according to Von Korff’s collaborative management of chronic illness care[34]. No definition of the BoT
Eton et al.	To finalize a conceptual framework of treatment burden	1st round: Semi-structured interviews in focus groups. Used Ritchie and Lewis Framework to create a conceptual framework 2nd round: Focus groups. To test the fitness of the framework and clarify new issues	Normalization Process Theory[35] and prior studies of treatment impact informed the questions. BoT defined as the workload of health care and its impact on patient functioning and well-being
Ridgeway et al.	To present the factors that patients with multimorbidity draw on to lessen perceptions of treatment burden	Same as Eton et al. Themes and subthemes were used to identify factors that mitigate treatment burden	Same as Eton et al.
Gallacher et al.	To assess the burden associated with treatment among patients living with chronic heart failure	Secondary analysis of qualitative interviews. Ritchie and Lewis framework analysis informed by Normalization Process Theory	Normalization Process Theory as a conceptual tool. BoT involves complex interactions between factors related to health care professionals and systems and factors related to patients’ characteristics
Kahn et al.	To explore the self-management strategies and treatment burden experienced by low income US primary care patients with chronic kidney disease	Semi-structured interviews one-on-one. Inductive thematic content analysis approach to analyze scripts and identify themes. Themes were reorganized in light of their direct application to Normalization Process Theory and treatment burden	Normalization Process Theory applied to chronic kidney disease. Treatment burden entails the patient’s engagement with providers, the health care system, their family or social support network, and personal self-care regimens
Tran, Montory et al.	To develop and validate an instrument for measuring treatment burden for patients with multiple chronic conditions	Semi-structured interviews one-on-one. The resulting measurement instrument was tested quantitatively on another group of patients	Three experts highlighted topics from a literature review. BoT defined as the impact of healthcare on patients’ functioning and well-being, apart from specific treatment side effects
Bayliss	To identify perceived barriers to self-care among patients with comorbid chronic diseases	Semi-structured interviews one-on-one. Used Qualitative Comparative Analysis to identify barriers to self-care	Interpreted the potential barriers to self-care that emerged from the analysis in light of the four components of chronic disease self-management. No definition of the BoT

The selection of meta-ethnography as method includes using the terms 1^st^ order interpretations (views of the participants), 2^nd^ order interpretations (interpretations of authors of included studies) and 3^rd^ order interpretations (new synthesis of 1^st^ and 2^nd^ order interpretations).

To answer research question A, components of the BoT were identified from the views of the participants in form of quotes (the 1^st^ order interpretations) and from authors’ listings of components. A grid was created, illustrating which studies each component was contained by, divided in categories found suitable (Table 3).

**Table 3:** Research question A: Components of BoT found in each study.

	[24]	[25]	[26]	[27]	[29]	[30]	[31]	[32]
**Interaction with the healthcare system**								
Spending time on travel and doctor visits	x	x	x	x	x	x	x	
Arranging appointments and transportation	x	x		x	x	x	x	x
Receiving contradictory advice	x	x	x	x	x	x		x
Attending multiple appointments	x	x	x		x		x	
Taking time off from work	x	x	x			x		
Administrating paperwork	x				x		x	
Waiting to obtain treatment	x	x	x					
Communicating with healthcare providers	x					x		x
**Medication burden**								
Coordinating medication	x	x	x	x	x	x	x	x
Medication interfering with other activities	x	x	x	x		x	x	
Suffering from side effects		x	x	x	x			x
Feeling stigmatized because of medication	x	x					x	x
Changing or obtaining prescriptions	x		x		x	x		
Using equipment or devices	x			x			x	
**Lifestyle changes**								
Altering diet: Cutting on foods or eating more	x		x	x	x	x	x	x
Planning and performing exercise	x		x	x	x	x	x	x
Quitting smoking	x		x			x		
**Financial burden**								
Paying for medication	x	x		x		x		x
Paying for health insurance or consultations	x	x		x		x		
**Learning about**								
Condition and treatment	x	x		x	x	x		x
Navigating in the healthcare system	x				x			
**Other**								
Self-monitoring of health status	x	x	x	x	x	x	x	
Relationship with friends and family: Being a burden	x	x	x	x	x		x	x

Step 4, *determining how the studies are related*: The 2^nd^ order interpretations regarding research question B were identified in the results and discussion sections of the papers. To answer research question C, the interpretations of the authors regarding the concept and nature of BoT were identified in the discussion sections.

Step 5, *translating the studies into one another*: The 2^nd^ order interpretations of the individual studies were compared within each research question using reciprocal translation. This means that findings and themes from the first study were compared to findings and themes from the second study, and individual and shared interpretations were identified. These findings were thereafter compared with findings from the third study and so on. Common conclusions were determined and both interpretations shared by multiple studies, and interpretations only identified in single studies were listed. Paraphrasing of the 2^nd^ order interpretations was used to list common contributions of studies, but an effort was made to keep the terminology from the original papers when possible.

Step 6, s*ynthesizing translations*: To develop new 3^rd^ order interpretations the core contributions of the 1^st^ and 2^nd^ order interpretations were identified and a “line of arguments” was created. This means listing the new 3^rd^ order interpretations providing a new understanding of the BoT. The Cumulative Complexity Model was used to provide an understanding of these interpretations.

Step 7, e*xpressing the synthesis*: The identified components of BoT were presented in the grid (Table 3) and discussed in the text. The 2^nd^ and 3^rd^ order interpretations considering question A and B were expressed in Table 4. Components, 2^nd^ and 3^rd^ order interpretations were discussed in the text supplemented by 1^st^ order interpretations in form of quotes.

**Table 4:** 2^nd^ and 3^rd^ order interpretations of research question B and C.

Research questions	2^nd^ order interpretations (authors’ interpretations)
Question B: Components of BoT attracting additional attention from patients with multimorbidi-ty	- Financial burden, lack of knowledge and the medication burden were the components found to be mentioned most often by patients[32] - Diet and exercise and frequent healthcare reminding patients of their health problem were ranked by patients as the most burdensome components[31] - Financial burden was most problematic for patients from Australia and low-income patients from the US[25, 30, 32] - Time and travel burden was particularly straining for patients living in remote locations[25] - The BoT was most straining when consequences of the treatment were not immediately visible for patients[24, 30] - Patients performed self-care tasks for conditions, in which they had an emotional investment, at the expense of other tasks[32]
Question C: Conceptuali-zation of burden of treatment in patients with multimorbidi-ty	- The BoT is described as being a multidimensional concept, with cyclical interrelated components[25, 27] - The BoT and the job of self-management was “hard work” for patients[27–30] - The work performed by the patients could be explained by the Normalization Process Theory[28–30] - The BoT consists of objective factors and the subjective experience[25] - The capacity of the patients is essential to their ability to manage the treatment burdens[24, 27, 28, 30]
	**3**^**rd**^ **order interpretations (synthesis)**
Overarching synthesis	- The BoT is a complex concept consisting of many different components, interacting with each other - The size of the experienced BoT is associated with the workload of demands, the capacity and the context - Socioeconomic deprived patients seemed to be more affected by certain burdens, especially the financial and time and travel burden - Patients seem to prioritize and synchronize demands to diminish the workload of the BoT - Included studies show how patients’ capacity is associated with their experienced BoT - Patients mobilize and coordinate resources to improve their ability to manage the BoT. - Routinizing the treatment work into daily life might be a way for patients to make workload and capacity fit together

Two of the included studies are based on the same interviews and focus groups (Eton et al. and Ridgeway et al.). But as the objective and results of the studies are different and do not overlap, both studies were included. The study conducted by Ridgeway et al. identifies factors that mitigate the BoT and does not focus on components of the BoT as the rest of the included studies. As a result the study is not included in the grid (Table 3).

### Quality appraisal

The included studies were rated with a 0 (not satisfying), ½ (partly satisfying) or 1 (satisfying) point for each of the 29 questions in Malterud’s checklist[23]. Missing points were most often due to lack of discussion of chosen data collection strategy, of content validity as well as discussion of rivaling explanations for the findings. As it appears in Table 1, all included studies scored >24 points equal to >84% of maximum points.

## Results

### Included studies

Due to the inclusion criteria nine qualitative studies were included. Fig 1 shows the selection process.

Five of the included studies were conducted in the US[26–28, 30, 32]. One study was undertaken across several, mainly Western, countries all over the world[24], while the three remaining were conducted in the UK[29], Australia[25] and France[31]. The population size varied from 16 to 1053 participants with a total number of 1367. The study populations showed variation in question of income (low/middle/high), areas (deprived in the city/wealthy/rural), ethnicity and age (middle-aged/older). Four studies recruited from primary care[26, 29, 30, 32]. Most studies conducted semi-structured interviews, but in a single study online questionnaires were analyzed qualitatively[24].

### Components of burden of treatment in patients with multimorbidity

The grid (Table 3) shows which components of the BoT were found in the included studies.

Patients with multimorbidity identified many different burdens in the **interaction with the healthcare system**. Among these, spending time on travel and doctor visits, arranging appointments and transportation as well as receiving contradictory advice were components most frequently found.

”For many appointments, you must leave time for: getting to the appointment, finding parking, waiting for the appointment, seeing the doctor, getting back home. That can easily wipe out a morning or an afternoon.”

[24]

Polypharmacy often resulted in a considerable **medication burden**. Patients had a hard time coordinating their medications. Some patients used helping devices to cope with this challenge, such as log books and dosette boxes.

“I’ve got a book and I note everything down. I note down when I’ve taken it, the dose, I note the time, the drug, and when I’ve taken it.”

[29]

Some patients felt a stigma related to taking medications while others were more concerned with the time spent on taking medications and the interference with other activities.

”I find my whole day is full of nothing but medicine.”

[26]

In the treatment of most conditions, **change of lifestyle** is one of the first suggestions of the healthcare provider. Patients were told to stop smoking, alter their diet and exercise more. This seemed to be a challenge to many patients suffering from more than one condition.

”Yeah, they (health care providers) tell me to try exercise or walking. But sometimes, I’ll be walking, and I be having pain in my legs.”

[27]

**Learning** to navigate in the healthcare system and finding information also posed a burden for patients. Especially in the first period of time after receiving the diagnoses, patients were burdened by the need to find information about their conditions and the treatment, not always knowing where to find that information.

“The only information that I get…is when you get these tablets in box form and they try and explain it to you …. Reading the leaflet, the print is that small and they use very big words for the likes of me—it’s foreign, I don’t know what it means.”

[29]

Other components often mentioned in the studies were the **financial burden**, the burden of self-monitoring of health status and the strain on relationships with friends and family.

### Components of BoT attracting additional attention from patients with multimorbidity (2^nd^ order interpretations)

Many of the included studies identified components or areas of the BoT to stand out, attracting additional attention from the patients in different ways. In one study the authors found the financial burden, lack of knowledge and the medication burden to be mentioned by most patients[32]. In another study patients were asked to rank the burden components and diet and exercise as well as frequent healthcare reminding patients of their health problem were ranked by patients as the most burdensome components[31].

Two of the included studies recruited participants from low-income areas in the US, and both reported socioeconomic burden among the most straining burdens[30, 32]. Patients found it hard to pay for public transportation, medication and insurance and to take time off from work to go and seek medical care. One patient could not afford to buy enough medicine for her hypertension and occasionally chose to do without it:

“Sometimes I go a month without taking it. And then I just crash and have, get dizzy, lightheaded, start getting sick, real bad headaches and stuff like that”

[30]

In many cases the amount of user charge and reimbursements from the state decided the financial strain on the patients.

“…tomorrow I’m going in to get … [treatment] and I don’t know how much we can claim back on that, it won’t be very much and it costs $200”

[25]

Many patients found it necessary to obtain a private health insurance, which was hard to afford especially for low-income earners and pensioners. Full time workers, however, experienced increased absence from work and potentially decreased income because of their treatments.

In the Australian study another group of patients were particularly exposed to the BoT[25]. Patients living in remote and rural locations experienced a considerable bigger time and travel burden than others. The need to travel up to 3–4 hours each way made accessing specialized care difficult. Some used their holidays from work for the travels, while others found it necessary to move closer to cities with the needed treatment options.

Furthermore, the cultural background of certain patients was found to add to the BoT as Aboriginals and Torres Strait Islanders pointed out structural racism in the healthcare system of Australia.

Patients wanted to be active partners in their own care[26, 28]. But one of the included studies, investigating patients with chronic kidney disease, showed that several patients didn’t understand the mechanisms of the treatment for their kidney disease[30]. They found it easier to comply with treatment for diseases they understood, like diabetes and hypertension, or specific approaches, like following a specific diet or exercise program. This was supported by another study finding that the BoT was experienced as most straining to the patients, when results or consequences of the treatment were not immediately visible[24]. The way patients thought and felt about the treatments had a substantial importance, and individuals were found to perform self-care tasks for conditions, in which they had an emotional investment at the expense of other tasks[32]. Patients were especially frustrated with their treatment, when the medication interfered with daily activities[25], while some patients felt no burden in treatment components already integrated in their lives[28, 31].

### Conceptualization of the burden of treatment (2^nd^ order interpretations)

Several of the included studies described the BoT as being a multidimensional concept, as it consisted of many different components burdening the patients. These components were found to be interrelated and connected in a cyclical way[25, 27]. Especially the financial burden had the potential to exacerbate other components, such as sticking to a diet.

“I live on vegemite sandwiches to keep my private health cover”

[25]

The included studies each had their own way to divide and categorize the components of the BoT. Some found three or four main themes[25, 27], one study created a taxonomy[24] and several studies fitted the components into an existing theory or model[24, 26, 29, 30].

The term”work” or”workload” was used to describe the BoT in several studies [27–30], in which the Normalization Process Theory was used to explain how patients performed that work and how it became embedded in everyday practice. The theory describes the work that patients do in four constructs. **Sense-making work** includes learning about treatments and their consequences. With **relationship work** patients engage with family members, friends or the general practitioner to help them manage their conditions. Patients have a demanding job on the daily basis, taking medication, attending appointments and thereby **enacting work**. **Appraisal work** means that patients spend time reviewing their treatment and deciding how to regulate it.

“If I’m going on a long trip on the bus, well I never take one (furosemide) in the morning because you have to keep going to the toilet, so if I’m going a long way, I miss the morning”

[29]

One study described the BoT as having an objective and subjective nature[25]. Objective factors such as number of conditions and medications, health status and time to access treatment were found to have an impact on the BoT, but so were factors determining the patient’s subjective experience like the patient’s educational level, financial resources, cultural background, age, gender and employment conditions. These individual differences were found to cause different treatment burdens for patients with similar treatment regimens.

Similar to these individual factors, several studies mentioned the capacity of the patients as central in their ability to manage the treatment burdens[24, 27, 28, 30]. Capacity refers to the patient’s cognitive and physical functioning, socioeconomic resources, family and social network, educational levels and literacy, cultural beliefs, and other factors. Furthermore the context, meaning the structure of healthcare and social support, was found important[24].

### 3rd order interpretations

The BoT in patients with multimorbidity shows to be a complex concept consisting of many different components, interacting with each other. The BoT has a cyclical nature, recurring in the Cumulative Complexity Model[13], which establishes the BoT both as a part of the workload but also as a feedback loop between patient health outcomes and workload.

The BoT seems to be strongly associated with the workload of treatment demands, determined by objective factors. This review shows that in order to manage this workload, some patients occasionally discuss with their healthcare provider, what part of the treatment they find most important. Other patients describe how they choose by themselves not to follow the most burdening parts of the treatment. In these ways, patients seem to prioritize between treatment modalities in order to synchronize demands, as described by the Cumulative Complexity Model. This might result in some patients not following prescribed treatments, they do not feel necessary, while other patients choose not to follow treatments they do not understand or treatments that do not provide a visible improvement or result.

“There is stuff that I am supposed to do, and stuff that I actually do. If I did everything I am supposed to do, my life would revolve around doctors and tests and such and there wouldn’t be very much left for living my life. So I’ve made a bunch of choices”

[24]

The studies included in this review all agree that the patient-experienced BoT is not only associated with the workload. Factors determining the capacity of the patients have been suggested to influence the patients’ subjective experience of the BoT. Two patients with similar diagnoses and treatments may differ in their capacity to handle their respective treatment burdens. The Cumulative Complexity Model provides an understanding, as it describes patient complexity as a balance between workload and capacity. From included studies this review has described how deprived socioeconomic status in some cases leaves low-income patients prone to burdens such as financial, time and travel burden. Another aspect of the capacity, the cultural background, might be responsible for patients feeling exposed to structural racism, making it harder for them to obtain healthcare.

As described in the Cumulative Complexity Model, patients might use strategies as mobilization and coordination, which might shape how capacity manifests. When patients manage to mobilize resources, whether it is their own abilities or support from family, their ability to handle the BoT is improved. It has been emphasized how coordination of medication is a substantial component of BoT, but coordination of resources, limitations and the environment might also be an important job for patients in order to improve their ability to manage the BoT. This is in alignment with the description of relationship work (cognitive participation) derived from The Normalization Process Theory, as relationships can be used as a resource.

“I would ask my wife, like because she worked in the medical field…”

[30]

Interactions between workload and capacity are described in the Cumulative Complexity Model. One of them, routinizing the treatment work into daily life seems to be a widely used strategy for patients.

“And so I have my alarm set to remind me to take that one [medication] […] because I’ll forget. I’ll get up and be like, okay, I gotta do this, this morning and I’ll forget. Yeah, so I set an alarm so I always remember “

[25]

As previously described, patients felt no burden in treatment components already integrated in their lives, as they had become daily routines.

Besides the capacity, another factor, the context, seems to be strongly related to the experienced BoT. The structure of the healthcare system is build up differently across the countries and in this review, patients from the US and Australia experienced a considerably bigger financial burden than patients from European countries, such as the UK and France. Other structural issues like access to specialized care and waiting time to see a doctor also seemed to be related to the size of the BoT. Furthermore, patients living in rural locations in Australia experienced a substantial time and travel burden, as specialized care is centralized to bigger cities.

## Discussion

This article is the first to systematically review and synthesize the existing qualitative data of the BoT experienced by patients with multimorbidity. It has provided an overview of components comprising the BoT, and of which of those components patients find to attract most attention. Furthermore, the factors associated with which burdens patients experience as most straining have been investigated. The BoT have been found to be a complex concept consisting of many different components interacting with each other. Workload, capacity and context have been shown to be associated with the size of the experienced BoT and strategies used by patients to alter the BoT have been revealed.

Overall, the contributions of the included studies showed a strong resemblance and were found complementary rather than conflicting. However, as seen in Table 3, none of the listed components, except for coordination of medication, were found in all of the included studies, and no studies contained all of the listed components. This can partly be explained by the lack of a common definition of the BoT. Another explanation is that the studies chose different ways to divide and present the components of the BoT.

The BoT is not static in time. “Learning about condition and treatment” was found to be an important component in most studies, but in one study[31] patients did not consider it a part of the BoT, as they had been living with their conditions for a long time. They had already adapted to it, integrated the knowledge about their conditions and treatments into the daily life, and did not mention it during the interviews.

### Strengths

Even though all included studies had the same purpose of investigating patient-experienced BoT in multimorbidity, the studies showed great heterogeneity regarding method and theoretical approach. This reflects the great complexity of this field and is seen as a strength, as the intention of this review was not to compare, but to synthesize and extend the knowledge of the BoT.

Another strength of this review is the great diversity among the included patients. While having multimorbidity in common, the participants showed great variation in income, education, origin, resident, ethnicity, cultural background and age, all adding to a broader description of the BoT.

The method used poses another strength. As described, the included studies approach the concept of BoT in different ways without contradicting each other. By using meta-ethnography, it was possible to synthesize the different approaches, descriptions and models into new 3^rd^ order interpretations while preserving the (1^st^ and 2^nd^ order) interpretations of the original studies.

All included studies did well in the quality assessment and reached medium to high quality in the evaluation. They all scored >84% of maximum points, when rated corresponding to satisfying answers to the checklist questions. One study failed to state the proportion of patients with multimorbidity in the study population[31]. But the fact that the participants on average consulted two different physicians and were treated with four different medications each day indicates a high proportion of patients with multimorbidity.

### Limitations

Recruitment method varied and not all studies accounted for the number of participants invited. Patients most burdened by illness as well as treatment might not have the time and energy to participate in voluntary research, and therefore overweight in participation of resourceful, less burdened patients is possible. Especially one study recruited some of their participants by advertising online and might have caused a selection of young, well-educated patients with access to a computer and skills to use it [24]. The study gathered answers from 1053 participants using an online questionnaire. This study is in line with the others regarding the concept of the BoT but also contributes with results showing that especially adherence problems, drug intake and time required for healthcare tasks are great burdens for patients with multimorbidity compared to patients with only one chronic condition.

The synthesis in this review is conducted using the Cumulative Complexity Model but other frameworks, as the Normalization Process Theory, might as well be suitable for conceptualizing the BoT.

The Normalization Process Theory describes the implementation and integration of new interventions and has shown useful to analyze and understand the BoT in stroke care[36]. It has in several studies included in this review been used as theoretical framework describing the patients’ treatment burden work in four constructs, as described under the 2^nd^ order interpretations.

Nonetheless the Cumulative Complexity Model was chosen in this review, as it was found as the best current model for understanding our focus, explaining the complexity of the multimorbid patients as well as their experience of the BoT. The model has not been tested empirically, but it provides a understanding of why a certain workload from the BoT seems to affect some patients more than others (factors of capacity and context). Furthermore the model outlines several mechanisms and strategies used by the patients in the included studies to avoid imbalance.

### Relation to other studies

Demain et al. have recently conducted a systematic review of qualitative research on treatment burden in long-term conditions[37]. They find that treatments and their total workload cause disruptions to a person’s biological, biographical and relational capacity. This seems to agree with findings in this review. The BoT component of “suffering from side effects” is similar to biological disruptions. Biographical disruptions describe the loss of freedom and the negative emotions equal to components of this review “spending time on travel and doctor visits”, “medication interfering with other activities” and “feeling stigmatized because of medication”. The component found in many studies included in this review, “being a burden in the relationship with friends and family”, is part of the relational disruptions described by Demain et al.

A certain amount of concordance is expected since three of the same studies are included in both reviews. However, Demain et al. also include studies investigating less common diseases like spasmodic dysphonia and tuberculosis as well as patients in terminal phase of renal disease of cancer. This review differs from the review of Demain et al. by focusing on patients with multiple, mainly common, conditions, who are not in the terminal phase.

### Perspectives

This review describes how patients with multimorbidity experience several burdens directly related to their healthcare provider. Providers need to improve their ability to communicate and to identify the individual level of the BoT. Discussing the BoT during consultations has proven to be a challenge to doctors treating patients with diabetes[38], while especially hospital, but also primary practice, doctors treating multimorbid patients find it hard to estimate the burden of their patients[31].

Experts have argued in favor of less disruptive treatment, ”minimally disruptive medicine,” as a solution to the problem of an increasing BoT[39]. To identify patients who would benefit from this approach, healthcare providers need to discuss components found in this review with their patients, but also considerations of capacity and context of the patient are crucial. This patient-centered care is in alignment with the “palliative approach”, which integrates values and principles from palliative care into the care of patients with chronic and potentially life-limiting conditions[40]. The aim of this approach is to achieve the highest possible quality of life for patients rather than focusing on the disease(s).

To help healthcare providers treat patients using these approaches, one improvement could be an initial screening of multimorbid patients with a tool measuring the BoT, before the beginning of a consultation. A measurement tool could also help determine which components of the BoT that are necessary to be discussed to help the patient. Furthermore it could be used to monitor effects of interventions on the BoT. Components found in this review could provide a framework for developing such a tool.

### Further research

Attempts to develop a tool to measure the BoT of patients with multimorbidity have already been done. One tool has been validated in a population of participants from 27 countries[41]. Another more comprehensive tool has recently been validated in the US, the Patient Experience with Treatment and Self-management (PETS)[42] developed from data of a qualitative study included in this review[27]. The tools include assessment of the financial burden, which may be more relevant in certain countries than others. Nonetheless the same tool might be used in several different countries given the strong resemblance in the BoT across the countries.

Future research should focus on linking a quantitative measure of BoT to clinical outcomes and investigate the outcomes of interventions. Conducting longitudinal studies of populations of multimorbid patients is one way to do this. That kind of study design would also allow researchers to investigate how the BoT changes over time, which no current studies show.

The capacity is a complex construct, which needs to be further explored. Work has been done to investigate the concept of capacity experienced by multimorbid patients[43] but further studies need to investigate the impact of the single elements of capacity on the patients’ experience of the BoT.

## Conclusions

This systematic review shows that the BoT experienced by patients with multimorbidity is a complex concept consisting of many different components interacting with each other. It is associated with the workload of demands, the capacity and the context. Patients use strategies such as prioritizing between treatments to diminish the workload and mobilizing and coordinating resources to improve their ability to manage the BoT. Patients try to routinize and integrate the treatment into their daily lives, which might be a way to maintain the balance between workload and capacity.

The components of the BoT occurring in different groups of multimorbid patients in different settings are identified. They seem to be interrelated and many components have the potential to attract additional attention from the patients. Which burden is experienced as the most straining by the single patient seems to be related to the objective factors of the workload, the capacity of the patient and the context. The financial burden and the time and travel burden seem to be experienced as particularly straining to patients with deprived socioeconomic status.

This investigation of the BoT may assist healthcare providers in facing the challenge of minimizing treatment burdens in patients with multimorbidity and provide a platform for further research. Especially quantitative studies are now needed to further develop the knowledge of the BoT.

## Supporting information

S1 PRISMA Checklist PRISMA checklist identifying how and where each element of the PRISMA process has been addressed in this paper. (DOC)

## References

[1] Ward BW, Schiller JS, Goodman RA. Multiple chronic conditions among US adults: a 2012 update. Preventing chronic disease. 2014;11:E62 doi: 10.5888/pcd11.130389 ; 24742395 10.5888/pcd11.130389 PMC3992293

[2] WHO USDoHaHSa. Global Health and Aging. 2011.

[3] Willadsen TG, Bebe A, Koster-Rasmussen R, Jarbol DE, Guassora AD, Waldorff FB, et al The role of diseases, risk factors and symptoms in the definition of multimorbidity—a systematic review. Scandinavian journal of primary health care. 2016;34(2):112–21. doi: 10.3109/02813432.2016.1153242 ; 26954365 10.3109/02813432.2016.1153242 PMC4977932

[4] Violan C, Foguet-Boreu Q, Flores-Mateo G, Salisbury C, Blom J, Freitag M, et al Prevalence, determinants and patterns of multimorbidity in primary care: a systematic review of observational studies. PloS one. 2014;9(7):e102149 doi: 10.1371/journal.pone.0102149 ; 25048354 10.1371/journal.pone.0102149 PMC4105594

[5] Barnett K, Mercer SW, Norbury M, Watt G, Wyke S, Guthrie B. Epidemiology of multimorbidity and implications for health care, research, and medical education: a cross-sectional study. Lancet. 2012;380(9836):37–43. doi: 10.1016/S0140-6736(12)60240-2 . 22579043 10.1016/S0140-6736(12)60240-2

[6] Fortin M, Lapointe L, Hudon C, Vanasse A, Ntetu AL, Maltais D. Multimorbidity and quality of life in primary care: a systematic review. Health and quality of life outcomes. 2004;2:51 doi: 10.1186/1477-7525-2-51 ; 15380021 10.1186/1477-7525-2-51 PMC526383

[7] Lee TA, Shields AE, Vogeli C, Gibson TB, Woong-Sohn M, Marder WD, et al Mortality rate in veterans with multiple chronic conditions. Journal of general internal medicine. 2007;22 Suppl 3:403–7. doi: 10.1007/s11606-007-0277-2 ; 18026809 10.1007/s11606-007-0277-2 PMC2219704

[8] Glynn LG, Valderas JM, Healy P, Burke E, Newell J, Gillespie P, et al The prevalence of multimorbidity in primary care and its effect on health care utilization and cost. Family practice. 2011;28(5):516–23. doi: 10.1093/fampra/cmr013 . 21436204 10.1093/fampra/cmr013

[9] De Geest S, Sabate E. Adherence to long-term therapies: evidence for action. European journal of cardiovascular nursing: journal of the Working Group on Cardiovascular Nursing of the European Society of Cardiology. 2003;2(4):323 doi: 10.1016/S1474-5151(03)00091-4 . 14667488 10.1016/S1474-5151(03)00091-4

[10] Haynes RB, McDonald HP, Garg AX. Helping patients follow prescribed treatment: clinical applications. Jama. 2002;288(22):2880–3. . 12472330 10.1001/jama.288.22.2880

[11] Ho PM, Rumsfeld JS, Masoudi FA, McClure DL, Plomondon ME, Steiner JF, et al Effect of medication nonadherence on hospitalization and mortality among patients with diabetes mellitus. Archives of internal medicine. 2006;166(17):1836–41. doi: 10.1001/archinte.166.17.1836 . 17000939 10.1001/archinte.166.17.1836

[12] Rasmussen JN, Chong A, Alter DA. Relationship between adherence to evidence-based pharmacotherapy and long-term mortality after acute myocardial infarction. Jama. 2007;297(2):177–86. doi: 10.1001/jama.297.2.177 . 17213401 10.1001/jama.297.2.177

[13] Shippee ND, Shah ND, May CR, Mair FS, Montori VM. Cumulative complexity: a functional, patient-centered model of patient complexity can improve research and practice. Journal of clinical epidemiology. 2012;65(10):1041–51. doi: 10.1016/j.jclinepi.2012.05.005 . 22910536 10.1016/j.jclinepi.2012.05.005

[14] Gallacher K, Jani B, Morrison D, Macdonald S, Blane D, Erwin P, et al Qualitative systematic reviews of treatment burden in stroke, heart failure and diabetes—methodological challenges and solutions. BMC medical research methodology. 2013;13:10 Epub 2013/01/30. doi: 10.1186/1471-2288-13-10 ; 23356353 10.1186/1471-2288-13-10 PMC3568050

[15] May CR, Eton DT, Boehmer K, Gallacher K, Hunt K, MacDonald S, et al Rethinking the patient: using Burden of Treatment Theory to understand the changing dynamics of illness. BMC health services research. 2014;14:281 doi: 10.1186/1472-6963-14-281 ; 24969758 10.1186/1472-6963-14-281 PMC4080515

[16] Noblit GW H R. Meta-ethnography: Synthesizing qualitative studies. Sage Publications 1988.

[17] Atkins S, Lewin S, Smith H, Engel M, Fretheim A, Volmink J. Conducting a meta-ethnography of qualitative literature: lessons learnt. BMC medical research methodology. 2008;8:21 doi: 10.1186/1471-2288-8-21 ; 18416812 10.1186/1471-2288-8-21 PMC2374791

[18] Ring N R K, Madava L, Jepson R. A guide to synthesising qualitative research for researchers undertaking health technology assessments and systematic reviews. NHS Quality Improvement Scotland. 2011.

[19] Pound P, Britten N, Morgan M, Yardley L, Pope C, Daker-White G, et al Resisting medicines: a synthesis of qualitative studies of medicine taking. Social science & medicine. 2005;61(1):133–55. doi: 10.1016/j.socscimed.2004.11.063 . 15847968 10.1016/j.socscimed.2004.11.063

[20] Campbell R, Pound P, Pope C, Britten N, Pill R, Morgan M, et al Evaluating meta-ethnography: a synthesis of qualitative research on lay experiences of diabetes and diabetes care. Social science & medicine. 2003;56(4):671–84. . 12560003 10.1016/s0277-9536(02)00064-3

[21] Moher D, Liberati A, Tetzlaff J, Altman DG, Group P. Preferred reporting items for systematic reviews and meta-analyses: the PRISMA statement. BMJ (Clinical research ed). 2009;339:b2535 doi: 10.1136/bmj.b2535 ; 19622551 10.1136/bmj.b2535 PMC2714657

[22] Salisbury C, Johnson L, Purdy S, Valderas JM, Montgomery AA. Epidemiology and impact of multimorbidity in primary care: a retrospective cohort study. The British journal of general practice: the journal of the Royal College of General Practitioners. 2011;61(582):e12–21. doi: 10.3399/bjgp11X548929 ; 21401985 10.3399/bjgp11X548929 PMC3020068

[23] Malterud K. Qualitative research: standards, challenges, and guidelines. Lancet. 2001;358(9280):483–8. doi: 10.1016/S0140-6736(01)05627-6 . 11513933 10.1016/S0140-6736(01)05627-6

[24] Tran VT, Barnes C, Montori VM, Falissard B, Ravaud P. Taxonomy of the burden of treatment: a multi-country web-based qualitative study of patients with chronic conditions. BMC medicine. 2015;13:115 Epub 2015/05/15. doi: 10.1186/s12916-015-0356-x ; 25971838 10.1186/s12916-015-0356-x PMC4446135

[25] Sav A, Kendall E, McMillan SS, Kelly F, Whitty JA, King MA, et al 'You say treatment, I say hard work': treatment burden among people with chronic illness and their carers in Australia. Health & social care in the community. 2013;21(6):665–74. Epub 2013/05/25. doi: 10.1111/hsc.12052 . 23701664 10.1111/hsc.12052

[26] Noel PH, Frueh BC, Larme AC, Pugh JA. Collaborative care needs and preferences of primary care patients with multimorbidity. Health expectations: an international journal of public participation in health care and health policy. 2005;8(1):54–63. doi: 10.1111/j.1369-7625.2004.00312.x . 15713171 10.1111/j.1369-7625.2004.00312.x PMC5060269

[27] Eton DT, Ridgeway JL, Egginton JS, Tiedje K, Linzer M, Boehm DH, et al Finalizing a measurement framework for the burden of treatment in complex patients with chronic conditions. Patient related outcome measures. 2015;6:117–26. Epub 2015/04/08. doi: 10.2147/PROM.S78955 ; 25848328 10.2147/PROM.S78955 PMC4383147

[28] Ridgeway JL, Egginton JS, Tiedje K, Linzer M, Boehm D, Poplau S, et al Factors that lessen the burden of treatment in complex patients with chronic conditions: a qualitative study. Patient preference and adherence. 2014;8:339–51. Epub 2014/03/29. doi: 10.2147/PPA.S58014 ; 24672228 10.2147/PPA.S58014 PMC3964167

[29] Gallacher K, May CR, Montori VM, Mair FS. Understanding patients' experiences of treatment burden in chronic heart failure using normalization process theory. Annals of family medicine. 2011;9(3):235–43. Epub 2011/05/11. doi: 10.1370/afm.1249 ; 21555751 10.1370/afm.1249 PMC3090432

[30] Kahn LS, Vest BM, Madurai N, Singh R, York TR, Cipparone CW, et al Chronic kidney disease (CKD) treatment burden among low-income primary care patients. Chronic illness. 2015;11(3):171–83. Epub 2014/11/25. doi: 10.1177/1742395314559751 ; 25416418 10.1177/1742395314559751 PMC4440843

[31] Tran VT, Montori VM, Eton DT, Baruch D, Falissard B, Ravaud P. Development and description of measurement properties of an instrument to assess treatment burden among patients with multiple chronic conditions. BMC medicine. 2012;10:68 Epub 2012/07/06. doi: 10.1186/1741-7015-10-68 ; 22762722 10.1186/1741-7015-10-68 PMC3402984

[32] Bayliss EA, Steiner JF, Fernald DH, Crane LA, Main DS. Descriptions of barriers to self-care by persons with comorbid chronic diseases. Annals of family medicine. 2003;1(1):15–21. doi: 10.1370/afm.4 ; 15043175 10.1370/afm.4 PMC1466563

[33] N W.L.. Social Research Methods: Qualitative and Quantitative Apporaches. 7th edn Pearson Education, Boston 2010;(I).

[34] Von Korff M, Gruman J, Schaefer J, Curry SJ, Wagner EH. Collaborative management of chronic illness. Annals of internal medicine. 1997;127(12):1097–102. . 9412313 10.7326/0003-4819-127-12-199712150-00008

[35] May CR, Mair F, Finch T, MacFarlane A, Dowrick C, Treweek S, et al Development of a theory of implementation and integration: Normalization Process Theory. Implementation science: IS. 2009;4:29 doi: 10.1186/1748-5908-4-29 ; 19460163 10.1186/1748-5908-4-29 PMC2693517

[36] Gallacher K, Morrison D, Jani B, Macdonald S, May CR, Montori VM, et al Uncovering treatment burden as a key concept for stroke care: a systematic review of qualitative research. PLoS medicine. 2013;10(6):e1001473 Epub 2013/07/05. doi: 10.1371/journal.pmed.1001473 ; 23824703 10.1371/journal.pmed.1001473 PMC3692487

[37] Demain S, Goncalves AC, Areia C, Oliveira R, Marcos AJ, Marques A, et al Living with, managing and minimising treatment burden in long term conditions: a systematic review of qualitative research. PloS one. 2015;10(5):e0125457 Epub 2015/05/30. doi: 10.1371/journal.pone.0125457 ; 26024379 10.1371/journal.pone.0125457 PMC4449201

[38] Bohlen K, Scoville E, Shippee ND, May CR, Montori VM. Overwhelmed patients: a videographic analysis of how patients with type 2 diabetes and clinicians articulate and address treatment burden during clinical encounters. Diabetes care. 2012;35(1):47–9. doi: 10.2337/dc11-1082 ; 22100962 10.2337/dc11-1082 PMC3241328

[39] May C, Montori VM, Mair FS. We need minimally disruptive medicine. BMJ (Clinical research ed). 2009;339:b2803 doi: 10.1136/bmj.b2803 . 19671932 10.1136/bmj.b2803

[40] Sawatzky R, Porterfield P, Roberts D, Lee J, Liang L, Reimer-Kirkham S, et al Embedding a Palliative Approach in Nursing Care Delivery: An Integrated Knowledge Synthesis. ANS Advances in nursing science. 2016 doi: 10.1097/ANS.0000000000000163 . 27930401 10.1097/ANS.0000000000000163 PMC5555976

[41] Tran VT, Harrington M, Montori VM, Barnes C, Wicks P, Ravaud P. Adaptation and validation of the Treatment Burden Questionnaire (TBQ) in English using an internet platform. BMC medicine. 2014;12:109 doi: 10.1186/1741-7015-12-109 ; 24989988 10.1186/1741-7015-12-109 PMC4098922

[42] Eton DT, Yost KJ, Lai JS, Ridgeway JL, Egginton JS, Rosedahl JK, et al Development and validation of the Patient Experience with Treatment and Self-management (PETS): a patient-reported measure of treatment burden. Quality of life research: an international journal of quality of life aspects of treatment, care and rehabilitation. 2017;26(2):489–503. doi: 10.1007/s11136-016-1397-0 . 27566732 10.1007/s11136-016-1397-0 PMC5753596

[43] Boehmer KR, Gionfriddo MR, Rodriguez-Gutierrez R, Dabrh AM, Leppin AL, Hargraves I, et al Patient capacity and constraints in the experience of chronic disease: a qualitative systematic review and thematic synthesis. BMC family practice. 2016;17:127 doi: 10.1186/s12875-016-0525-9 ; 27585439 10.1186/s12875-016-0525-9 PMC5009523

